# Degradome, small RNAs and transcriptome sequencing of a high-nicotine cultivated tobacco uncovers miRNA’s function in nicotine biosynthesis

**DOI:** 10.1038/s41598-020-68691-y

**Published:** 2020-07-16

**Authors:** Jingjing Jin, Yalong Xu, Peng Lu, Qiansi Chen, Pingping Liu, Jinbang Wang, Jianfeng Zhang, Zefeng Li, Aiguo Yang, Fengxia Li, Peijian Cao

**Affiliations:** 1 China Tobacco Gene Research Center, Zhengzhou Tobacco Research Institute of CNTC, Zhengzhou, 450001 China; 2 China Tobacco Science and Technology Information Center, Zhengzhou Tobacco Research Institute of CNTC, Zhengzhou, 450001 China; 3 Tobacco Research Institute, Chinese Academy of Agricultural Sciences, Qingdao, 266101 China

**Keywords:** Plant sciences, Plant biotechnology

## Abstract

Tobacco (*Nicotiana tabacum*) is considered as the model plant for alkaloid research, of which nicotine accounts for 90%. Many nicotine biosynthetic genes have been identified and were known to be regulated by jasmonate-responsive transcription factors. As an important regulator in plant physiological processes, whether small RNAs are involved in nicotine biosynthesis is largely unknown. Here, we combine transcriptome, small RNAs and degradome analysis of two native tobacco germplasms YJ1 and ZY100 to investigate small RNA’s function. YJ1 leaves accumulate twofold higher nicotine than ZY100. Transcriptome analysis revealed 3,865 genes which were differently expressed in leaf and root of two germplasms, including some known nicotine and jasmonate pathway genes. By small RNA sequencing, 193 miRNAs were identified to be differentially expressed between YJ1 and ZY100. Using in silico and degradome sequencing approaches, six nicotine biosynthetic genes and seven jasmonate pathway genes were predicted to be targeted by 77 miRNA loci. Three pairs among them were validated by transient expression in vivo. Combined analysis of degradome and transcriptome datasets revealed 51 novel miRNA-mRNA interactions that may regulate nicotine biosynthesis. The comprehensive analysis of our study may provide new insights into the regulatory network of nicotine biosynthesis.

## Introduction

Around 20% of plants are known to produce alkaloids, a diverse class of more than 12,000 different N-containing natural product compounds which are important for plant defense and medical use^1,2^. Cultivated tobacco (*Nicotiana tabacum*), as a natural allotetraploid, is possibly evolved from hybridization of two diploids *N. tomentosiformis* and *N. sylvestris*^3,4^. Nicotine accounts for around 90% of total alkaloids in tobacco, which made tobacco as a model plant to study the biosynthesis, transportation, accumulation, and degradation of alkaloids. Extensive studies have established that nicotine is produced in roots before being transported to and accumulating in leaves^5–7^. Nicotine functions as a defense toxin to many insect herbivores^8^. Mechanical wounding and wounding-elicited jasmonate signaling are known to increase the accumulation of nicotine^9–11^. For instance, substantially increased nicotine synthesis and accumulation is known to result from the agricultural practice known as topping, wherein the early developing inflorescence organs are removed by producers to prevent seed production^12,13^.

Nicotine is synthesized from putrescine, which is derived from arginine or ornithine^14^. Most of the enzymes involved in this pathway have been identified and characterized in the course of many decades of research, including ornithine decarboxylase (*ODC*), putrescine N-methyltransferase (*PMT*), aspartate oxidase (*AO*), quinolinate synthase (*QS*), quinolinate phosphoribosyl transferase (*QPT*), N-methylputrescine oxidase (*MPO*), and berberine bridge enzyme-like (*BBL*), among others^4,6,14^. There has also been extensive research about the genetic control of nicotine biosynthesis, and these researches have highlighted particularly large regulatory impacts from two transcription factors families: APETALA2 (*AP2*)/ethylene response factors (*ERF*) and MYC2-like basic helix-Loop-helix (*bHLH*)^15–18^. These studies have collectively established a rich set of resources which could be called “nicotine module” for understanding both the genetic and biochemical basis of nicotine biosynthesis and for modeling the uptake, transportation, and accumulation of this classic plant defense compound^4^. In addition to these canonical pathways, a recent study found that tobacco microRNA (miRNA) nta-eTMX27 could target *QPT2* transcripts and subsequently affects nicotine content in tobacco, indicating the role of small RNAs in nicotine biosynthesis^19^.

MiRNAs, one of small noncoding RNA, are comprising 21–24 nucleotides^20^. They have essential functions in plant such as development, growth, maturation, cell differentiation, and response to various abiotic and biotic stresses^21–24^. Recent studies have found that miRNAs play important roles in regulation of natural product biosynthesis in plants. In one example, miR393 indirectly regulates natural product biosynthesis by regulating auxin signaling^25^, and some miRNAs, including miR156, miR165 and miR166, have been revealed to regulate anthocyanin biosynthesis in Arabidopsis and *Solanum Lycopersicum*^26–29^. As development of sequencing techniques and improvement of tobacco genome^30–33^, several studies have used small RNA sequencing to profile miRNAs in tobacco^12,13,34–38^. These studies have revealed insights including characterization of five miRNA families that exhibit differential accumulation profiles under drought conditions^39^, and a functional demonstration that miR164 targets mRNA transcripts of NtNAC-R1 and that such targeting increases upon topping and leads to multiple root-related phenotypes^40^.

Although we can use bioinformatics approaches to predict miRNA targets, the functional verification is time–cost^41–44^. Recently, degradome sequencing, is considered as a powerful approach that connects high-throughput sequencing and screen targets for miRNA/ta-siRNA (trans-acting siRNA) in a large scale^41,42^. High-throughput sequencing of the cDNA and degradome sequencing can provide rich resource about transcripts that undergo degradation. By degradome sequencing, targets for many miRNA have been identified. Meanwhile, the accurate pairing information between miRNA and its degradation fragments in plants (for example: Arabidopsis, rice, soybean, and cotton) has been determined^41–46^.

In this study, we aimed to investigate small RNA-mediated nicotine biosynthesis in tobacco plants. Two tobacco germplasms, YJ1 and ZY100, which showed different nicotine accumulation in leaves were employed. We inferred that the regulation or biosynthetic pathway of nicotine may differ between these two germplasms. To this end, comparison of transcriptomes by using leaf and root samples of two germplasms was performed. We asked if some new genes and miRNAs were differently expressed. By degradome and in silico analysis, we firstly identified miRNAs that target to known nicotine module genes. Several miRNA-mRNA pairs were confirmed by GFP cleavage assays. Combining the transcriptome comparison and degradome datasets, we asked if there are some novel miRNA-mRNA pairs which may be involved in nicotine metabolism. Our results showed that 77 miRNAs could target to known nicotine module genes, and predicted 51 novel miRNA-mRNA target pairs as the candidates of nicotine regulators.

## Results

### Transcriptome profiling in low and high nicotine germplasms

**Figure 1:**
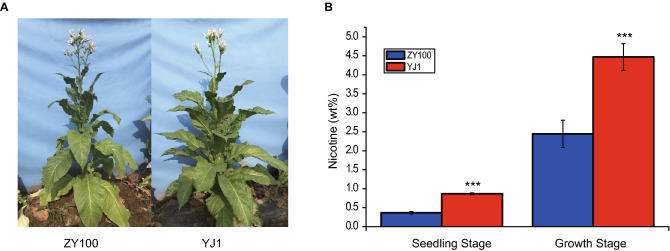
Phenotype for ZY100 and YJ1. (**A**) Phenotype for ZY100 and YJ1 at mature stage. (**B**) Nicotine level of leaves for ZY100 and YJ1 at different developmental stages.

ZY100 and YJ1 were two common used tobacco germplasms and stored in National Crop Germplasm Resource Center of China. There was no growth difference between ZY100 and YJ1 (Fig. 1A). However, YJ1 accumulated more than two folder higher nicotine in the leaves of different stages than ZY100, suggesting the regulation and biosynthesis of nicotine may differ between these two germplasms (Fig. 1B). To identify nicotine-biosynthesis-related genes and regulation network, transcriptomes of twelve samples from leaf and root of ZY100 and YJ1 were sequenced (Table 1). There were around 50 million paired-end reads for each library (Table 1). On the average, 98.07% of the clean reads could be mapped to the tobacco genome (Table 1). Using FPKM, we found 35,691 protein-coding genes (> 1 FPKM) were expressed in at least one sample (Supplementary Data set S1).

**Table 1:** Summary of transcriptome and small RNA sequencing data generated for 12 samples using Illumina sequencing platform.

Germplasm	Tissue	ID	Transcriptome sequencing		Small RNA sequencing		
			Clean reads	Map rate (%)	Clean reads	MiRNA	Known miRNA
YJ1	Leaf	YL_1	44,639,048	99.19	16,654,549	1,382	301
		YL_2	50,233,183	99.11	18,910,841	1,438	311
		YL_3	59,383,213	99.71	22,173,028	1,490	313
	Root	YR_1	48,414,106	97.2	17,590,031	1,339	316
		YR_2	52,581,326	95.48	13,508,800	1,203	305
		YR_3	47,854,575	98.1	19,049,339	1,428	311
ZY100	Leaf	ZL_1	51,093,565	98.41	19,983,272	1,428	317
		ZL_2	46,752,230	98.24	21,845,215	1,499	315
		ZL_3	35,146,777	98.03	20,268,505	1,459	314
	Root	ZR_1	40,234,431	97.86	19,205,924	1,395	308
		ZR_2	42,423,588	98.18	18,092,298	1,405	311
		ZR_3	52,897,023	97.32	21,706,134	1,468	313

**Figure 2:**
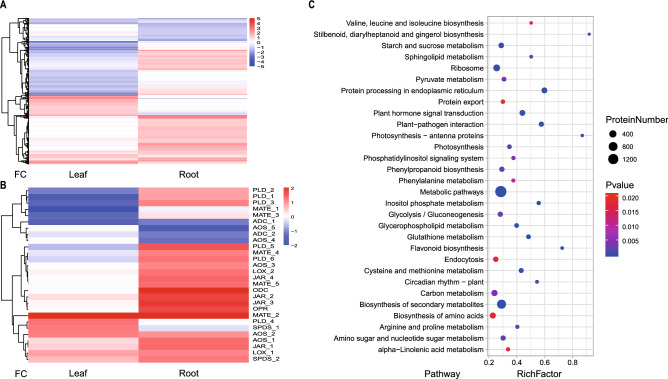
Expression pattern of protein-coding genes by RNA-Seq. (**A**) Hierarchical cluster analysis of all DEGs in leaf and root. (**B**) Hierarchical cluster analysis of known DEGs involved in nicotine biosynthesis. (**C**) KEGG pathway enrichment analysis for all DEGs. *FC* fold change, X-axis represents fold change between two germplasms.

To identify candidate nicotine-regulation-related genes, we conducted a transcriptome comparison analysis between ZY100 and YJ1. Totally, 1,832 differential expressed genes (DEG) from leaf and 2,216 DEGs from root were identified (Fig. 2A and Supplementary Data set S1). Nicotine is known to be synthesized in the root of tobacco plants. The expression of *ODC*, a nicotine biosynthetic gene, was significantly up-regulated in the root of YJ1. In addition, the transcription levels of three nicotine transporters, *MATE* (multidrug and toxic compound extrusion) family genes were also increased in the root of YJ1 compared with that in ZY100 (Fig. 2B). Interestingly, a number of jasmonate biosynthetic genes (e.g., *AOS* (Allene oxide synthase), *LOX* (linoleate 9S-lipoxygenase), and *JAR* (Jasmonoyl–L-amino acid synthetase)) were also up-regulated both in the root and leaf of YJ1. Kyoto Encyclopedia of Genes and Genomes (KEGG)^47^ and gene ontology (GO) analysis revealed that the DEGs were enriched in primary and secondary metabolic pathways (Fig. 2C and Supplementary Fig. S1). For example, arginine and proline metabolism pathway was significantly enriched, whereas arginine could be broken down into ornithine, a substrate of nicotine biosynthesis. Moreover, the DEGs were also preferentially involved in plant hormone signal transduction and circadian rhythm, which are known to regulate nicotine biosynthesis^48^.

### Known and novel miRNA identification

**Figure 3:**
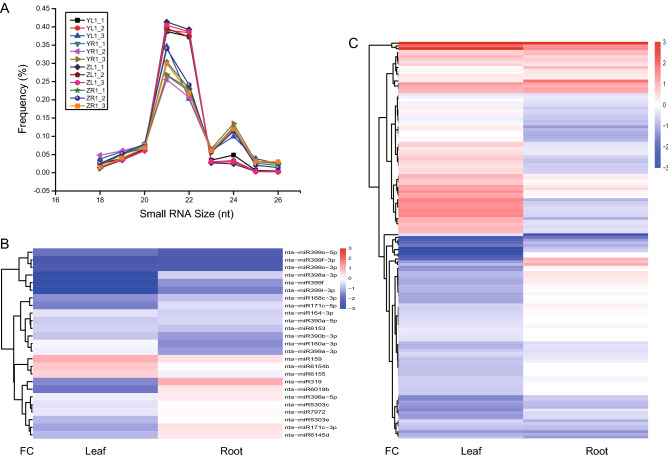
Expression pattern of conserved and novel miRNAs. (**A**) Length distribution of the small RNAs in different libraries. (**B**) Hierarchical cluster analysis of known miRNAs. (**C**) Hierarchical cluster analysis of novel miRNAs. FC fold change, X-axis represents fold change between two germplasms.

To characterize the small RNA accumulation patterns in tobacco, we constructed small RNA libraries using the same samples in our transcriptome analysis. Totally, we obtained 228 million clean reads among all libraries, with around 20 million for each library (Table 1). Size distribution analysis of reads between 18 and 26 nucleotides (nt) in length revealed that the most of small RNAs in our samples ranged from 18–24 nt (Fig. 3A). We detected two peaks at 21 and 24 nt in most samples, indicating that small RNAs of 21 and 24 nt are the two major size classes in tobacco, consistent with a previous study in this species^12,13,34,39^ and with reports for Arabidopsis and rice^49,50^.

After removing small RNA reads originating from other small non-coding RNAs (including tRNA, rRNA, snRNA, and snoRNA), the left reads were aligned to the tobacco reference genome^30^. Notably, pairwise analysis of the correlation in expression patterns between sample replicates indicated that the small RNA sequencing data was of good quality (Supplementary Fig. S2). Candidate miRNA-producing genomic loci were then identified by miR-PREFeR^51^, and revealed a total of 1,967 potential such loci from the data for the twelve libraries, each of which had corresponding mature and star sequences (Table 1, Supplementary Data set S2).

We next used miRbase v21 to identify the evolutionarily conserved miRNAs in our tobacco Data set^52^. This stringent, BLAST-based analysis allowed no more than one mismatch with a putative homolog sequence in miRbase. This analysis identified a total of 321 candidate conserved miRNA-producing genomic loci (representing a total of 82 conserved candidate miRNA species), which were grouped into 47 miRNA families (Table 1, Supplementary Data set S2). Among these 47 conserved families, the most abundant miRNA families (nta-miR396, 159, 166) whose expression levels were > 2,500 transcripts per million (TPM), are conserved among bryophytes, monocots, and eudicots (Supplementary Table S1, Supplementary Data set S2). The remaining 1,646 candidate miRNA-producing genomic loci (representing 1,224 non-redundant, detected miRNA species in tobacco) were therefore classified as novel miRNAs (Supplementary Data set S2; See Supplementary Table S2 for the top ten most abundant candidates, whose normalized expression levels were > 500 TPM clean tags).

### Expression pattern of conserved and novel miRNAs

After identifying miRNA loci, we further characterized miRNA expression patterns in leaf and root of ZY100 and YJ1. The miRNA candidates, detected more than 3 TPM at least in one library, were used to check the expression pattern. Cluster analysis of differentially expressed miRNAs was based on fold change (log2) between ZY100 and YJ1 for leaf and root samples. Totally, 46 differentially expressed known miRNA loci and 147 differentially expressed novel miRNA loci were identified for all samples (Fig. 3B,C and Supplementary Data set S2). Moreover, the number of down-regulated miRNA loci were much higher than up-regulated candidates (Fig. 3B,C). Interestingly, the number of down- and up-regulated DEGs showed a contrary pattern (Fig. 2A,B), suggesting the existence of miRNA-mRNA interaction pairs.

### Target identification by in silico and degradome analysis

Usually, miRNAs and their targets have perfect complementarity sequence in plants, which allows the prediction of targets by in silico tools. By psRobot, we have identified 44,267 targets for these 1,320 miRNA loci. Further, using degradome sequencing^53^, a total of 29,499,583 and 25,405,269 reads for leaf samples of ZY100 and YJ1 were generated (Table 2). More than 65% of the reads could be mapped to the tobacco cDNA database^30^. On average, 869 targets were identified with cutoff of P-value <  = 0.05.

**Table 2:** Summary data of degradome sequencing from ZY100 and YJ1.

	YJ1	ZY100
	1: before topping(D1)	1: before topping (D4)
Number of reads	25,405,269	29,499,583
Map rate	16,763,004 (65.98%)	19,843,429 (67.27%)
Number of targets	34,267	37,327
Targets (p < = 0.05)	781	957
**Categories**		
0	426 (54.5%)	579 (60.5%)
1	152 (19.5%)	146 (15.3%)
2	81 (10.4%)	102 (10.7%)
3	67 (8.58%)	74 (7.73%)
4	55 (7.04%)	56 (5.86%)

Moreover, the maximum cleavage sites for each sample were category 0 (mean = 57.52%) and minimum was category 4 (mean = 6.45%) (Table 2). These cleavage sites for each target were shown in the target plots (T-plots) (Supplementary Fig. S3). Finally, using degradome sequencing, 1,306 targets for a total of 538 miRNA loci (152 known and 386 novel) were identified.

Combing in silico and degradome approach, 45,373 targets were revealed for most of (79%) miRNAs. A major group of the conserved miRNA targets (3,850 miRNA target pairs) were transcription factors, including MYB, NAC, SBP-box, bZIP and AP2 (Table 3). Compared with previous identified targets, our study can clearly show the cleavage products for miRNA targets, including miR156-SBP (Supplementary Fig. S3a), miR164-NAC (Supplementary Fig. S3c), miR172-bHLH (Supplementary Fig. S3e) and miR172-ERF (Supplementary Fig. S3g). Moreover, their expression pattern is also highly correlation (Supplementary Fig. S3b,d,f,h). To some extent, it means the reliability of our result.

**Table 3:** Overview of transcription factor targets for known miRNA candidates.

MiRNA	Target	MiNRA	Target	MiRNA	Target
nta-miR156	SBP	nta-miR5303	GRF	nta-miR7997c	HB
nta-miR159	MYB		BBR/BPC		bHLH
nta-miR160	ARF		C3H		G2-like
	HSF		CCAAT		MYB
nta-miR164	NAC		FAR1		GRF
nta-miR166	HB		G2-like		SBP
nta-miR167	ARF		GeBP		TUB
nta-miR171	GRAS		HB		WRKY
nta-miR172	AP2-EREBP		MADS		MADS
	bHLH		MYB		C3H
nta-miR319	TCP		NAC		
	MYB		RWP-RK		
nta-miR396	GRF		SBP		
nta-miR399	CCAAT		TUB		
nta-miR6020b	G2-like		WRKY		

### Identification of nicotine module gene-miRNA interaction pairs

**Figure 4:**
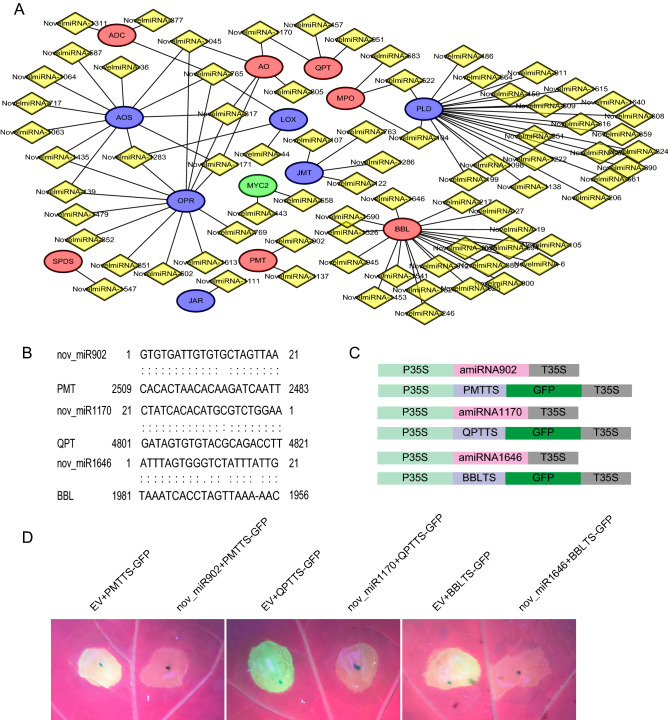
The miRNA-mRNA coexpression network for known nicotine biosynthesis genes. (**A**) The coexpression network for known nicotine biosynthesis genes. (**B**) Target site sequence between miRNA and their corresponding targets. (**C**) Overexpression vectors constructed for transient expression system in tobacco. (**D**) Co-infiltrated leaves were photographed at the third day after infiltration under UV light. Yellow color represents miRNA candidates; Red color represents known genes involved in nicotine biosynthesis pathway; Green color represents known transcription factors regulating nicotine biosynthesis; Blue color represents known genes involved in JA pathway.

We examined the loci of known nicotine module genes by aforementioned constructed network, and identified 77 miRNAs targeting to 6 nicotine and 7 jasmonate biosynthetic genes (Fig. 4A). Some of the miRNAs have more than one targets, such as a novel miRNA1283 could target three jasmonate biosynthetic genes. On the other hand, some of the genes could be targeted by many miRNAs, e.g. *BBL*. We also identified two miRNAs could target to the main regulator of nicotine biosynthesis, *MYC2*. To verify the constructed subnetwork, three pairs of miRNA-mRNA targets (nov-miR902-PMT, nov-miR1170-QPT and nov-miR1646-BBL) were selected and validated in tobacco using transient expression platform (Fig. 4B). We constructed overexpression vectors of nov-miR902, nov-miR1170 and nov-miR1646 (Fig. 4C).

Target sites of miRNA in PMT, QPT, and BBL were inserted into a GFP gene over-expressed vector, respectively (Fig. 4C). According to the configurations, agrobacterium tumefaciens infiltration (GV3101) was used for co-expression of GFP gene carrying target site and miRNAs. As shown in Fig. 4D, nov-miR902, nov-miR1170, and nov-miR1646 could target their corresponding genes and decreased the expression of GFP obviously, indicating the reliability of our predicted network.

### Identification of novel miRNA-mRNA pairs involved in nicotine metabolism

**Figure 5:**
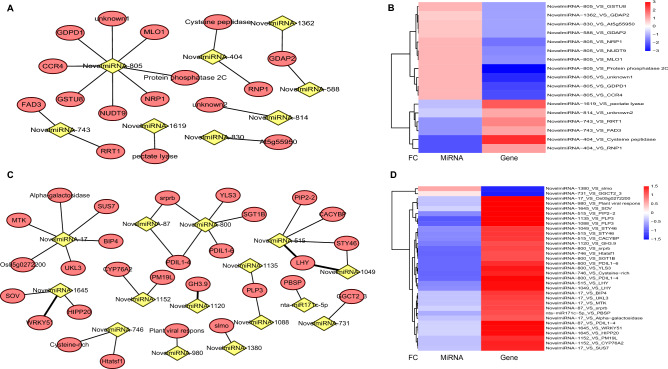
Novel miRNA-mRNA interaction network involved in nicotine metabolism. (**A**) Novel miRNA-mRNA interaction network in leaf. (**B**) Heatmap for mRNA and miRNA interaction pairs in leaf network. (**C**) Novel miRNA-mRNA interaction network in root. (**D**) Heatmap for mRNA and miRNA interaction pairs in root network. Yellow color represents miRNA candidates; Red color represents genes; *RNP1* Heterogeneous nuclear ribonucleoprotein 1, *GDPD1* Glycerophosphodiester phosphodiesterase, *CCR4* Serine/threonine-protein kinase-like protein, *RRT1* Rhamnogalacturonan I rhamnosyltransferase 1, *NRP1* Nodulin-related protein 1, *FAD3* Omega-3 fatty acid desaturase, *NUDT9* Nudix hydrolase 9, *MLO1* Nudix hydrolase 9, *PLP3* Patatin-like protein 3, *PDIL* Protein disulfide isomerase-like, *LHY* Late elongated hypocotyl and circadian clock associated-1-like, *srprb* Signal recognition particle receptor subunit beta, *Htatsf1* HIV Tat-specific factor 1 homolog, *STY46* Serine/threonine-protein kinase, *CACYBP* Calcyclin-binding protein, *GH3.9* Putative indole-3-acetic acid-amido synthetase, *SUS7* Sucrose synthase 7, *GGCT2;3* Gamma-glutamylcyclotransferase 2–3, *MTK* Methylthioribose kinase, *HIPP20* Heavy metal-associated isoprenylated plant protein 20.

To search the novel genes and miRNAs which may regulate nicotine biosynthesis, we combined the transcriptome, small RNAs and degradome dataset. Genes and miRNAs showed differentially expressed in YJ1 and ZY100 were selected, and were paired based on the constructed network. Totally, 51 miRNA-mRNA interaction pairs were annotated. Among them, 17 pairs were identified in leaf and 34 pairs were identified in root (Fig. 5A,C). Based on the fact that nicotine levels were differently accumulated in two germplasms, we inferred that these novel pairs may be involved in nicotine biosynthesis and regulation. Most of identified genes in root were up-regulated in YJ1 compared with ZY100 (Fig. 5D); while down-regulated genes in YJ1 were more abundant in the leaf samples (Fig. 5B). Giving that root tissue is the factory of nicotine, these miRNA-mRNA pairs in root may directly regulate nicotine biosynthesis. For example, WRKY51 in Arabidopsis was known to regulate jasmonate biosynthesis^54^. We found the transcription level of WRKY51 gene was higher in the root of YJ1 than that in ZY100. The GH gene family are amido synthetases which conjugate free auxin or jasmonic acid with amino acids to form the bioactive phytohormone^55^. A GH gene, GH3.9, was also up-regulated in the root of YJ1. Moreover, the circadian clock gene LHY showed similar pattern like WRKY51 and GH3.9. In wild tobacco, the core circadian clock ZTL protein is known to regulate nicotine biosynthesis through JA signaling^48^. We proposed that LHY may have similar function in cultivated tobacco.

## Discussion

In recent years, the availability of diverse germplasms and high-throughput sequencing platform provided an opportunity to study secondary metabolism. Although many genes involved in nicotine biosynthesis and regulation have been reported, the studies about small RNA’s role are elusive^56^. In our work, three important high-throughput methods, namely, small RNA, degradome and transcriptomics sequencing were applied to investigate mechanisms of nicotine biosynthesis in tobacco. Giving that nicotine is highly accumulated across all of the tissues in tobacco plant, it could be considered as a constitutively defense, although mechanical wounding can induce a higher nicotine level^8,9^. There is large difference in the constitutive level of nicotine in the leaves between YJ1 and ZY100, which are useful tools for investigating nicotine biosynthetic pathway. By transcriptome analysis, a total of 3,865 genes and 193 miRNAs whose transcription levels showed significant difference between ZY100 and YJ1 were identified. We focused on these genes and miRNAs that have contrary pattern. Combining transcriptome and degradome analysis, we finally identified 51 pairs as new regulators in the nicotine metabolism. Meanwhile, we identified 77 miRNAs targeting to known nicotine biosynthesis and regulation pathway genes.

Based on the omics data, GH3.9 and WRKY51 that may be involved in jasmonate acid (JA) signaling were predicted as potential regulators in nicotine biosynthesis. Both genes were up-regulated in YJ1 and their associated miRNAs were down-regulated. Of them, GH3.9 may be a JA biosynthetic gene; while WRKY51 may function upstream of JA biosynthesis^54^. Moreover, the transcriptome comparison revealed many other JA biosynthetic genes had higher transcription levels in YJ1 compared with ZY100 (Fig. 2B). Considering nicotine biosynthesis was regulated by JA pathway, the high nicotine level in YJ1 may be caused by the activation of JA biosynthesis. Meanwhile, we indeed found the jasmonic acid level were higher in YJ1 than ZY100 (Supplementary Fig. S4). To date, only one miRNA, miR319, is known to target TCP transcription factors which in turn regulate JA biosynthesis^57,58^. Our new identified miRNAs that associated with JA signaling pathway are also potential interesting.

Li et al. found nta-eTMX27 could regulate nicotine biosynthesis by targeting QPT2^19^. However, this candidate is not found by our study. One explanation could be the different genome used in different studies. Alternatively, we identified 36 miRNAs targeting to 6 nicotine biosynthetic genes. Three miRNA-mRNA pairs (nov-miR902-PMT, nov-miR1170-QPT and nov-miR1646-BBL) were validated to be involved in regulating nicotine biosynthesis genes by transient expression in vivo (Fig. 4). Further experiments by stable over-expression of these miRNAs or target-mimicry approach to block the function of them are required to investigate their roles in nicotine biosynthesis^58,59^. In summary, we revealed miRNA-mediated regulatory network of nicotine biosynthesis in tobacco plant. These newly identified candidates are valuable tools to dissect nicotine pathway in the future. Meanwhile, this study will enrich the understanding of small RNA’s function in regulating plant secondary metabolites.

## Methods

### Plant materials collection

The *Nicotiana tabacum* germplasms ZY100 and YJ1 were used in this study. These two germplasms were cultivated in an experimental field (Qingdao, Shandong, China) using the typical agricultural practices for tobacco in this production area. Then, leaf and root tissues were collected for these two germplasms at mature growth stage. The leaf from middle positions (no. 11–13) was harvested. Three plants were collected as a replicate, and six independent replicates were taken for analysis. After harvest, the samples were immediately frozen in liquid nitrogen and stored at − 80 °C for RNA extraction. Total RNA was extracted from the collected tissues similar with methods reported in Jin et al.^60^.

### RNA-Seq, small RNA, and degradome sequencing

The RNA-Seq sequencing was paired end reads using Illumina HiSeq 2,500 platform. For the small RNA (sRNA) sequencing, libraries were using Illumina HiSeq 2,500 platform for 50 bp single end reads. For the degradome libraries, single end sequencing (50 bp) was performed with an Illumina HiSeq 2,500 platform. These libraries were constructed similarly with previously studies by German et al.^53^.

### RNA-sequencing data analysis

Trimmomatic (v0.30) was used to remove the adaptor sequences and the low quality reads of RNA-Seq reads. Then, left reads were mapped to reference genome of tobacco^30^ with bowtie2^61^. Gene expression levels were assessed using FPKM (fragments per kilo bases per million reads) values. To define differentially expressed mRNA transcripts, we used P-value ≤ 0.05 (T–test) and more than twofold change as cutoff.

### The sRNA Data process

Trimmomatic (v0.30) was used to remove the adaptor sequences of the sRNA reads. The remaining clean reads were mapped to the reference genome of tobacco using bowtie^61^. In order to remove reads originating from tRNA, rRNA, and snRNA, these high quality reads were also mapped to the Rfam^62^ database with BLASTN. The reads mapping to Rfam were discarded. The miRNA candidates were predicted using miR-PREFeR^51^.

### Target identification by degradome sequencing

The reads mapping to the tobacco cDNA were used to identify miRNA cleavage sites by CleaveLand^63^. The cleavage sites were defined as significant with cutoff P-value ≤ 0.05. Commonly, based on the reads abundance, the identified cleavage sites were group into five different categories (0–4). Category 4 with minimum confidence has only one read supporting the cleavage site, whereas categories 0–3 always have more than one reads.

### Nicotine level quantization

Quantification of the nicotine levels followed standard methods^60^. Briefly, freeze-dried leaf tissues were ground to a uniform powder and stored at − 80 °C. The nicotine levels of YJ1 and ZY100 germplasm were measured in six biological samples. First, 2.5 mL 5% sodium hydroxide solution were added to 0.3 g of tobacco powder, and added 20 mL 0.01% triethylamine/methyl tert-butyl ether solution after 15 min, with ultrasonic extraction for 15 min at room temperature. After 6000 rpm and 5 min centrifugation, 2 ml organic phase was prepared for GC–MS based detection.

### Validation of miRNA target interaction in vivo

To validate interaction between miRNA and targets in vivo, using agrobacterium tumefaciens infiltration (GV3101), we express miRNA and its corresponding targets fused with a GFP reporter gene using leaf tissue. Then, we preformed similar transient expression experiment with previous work^64^.

### Co-expression network visualization

The mRNA and miRNA interaction was obtained by in silico and degradome analysis. Subsequently, these interaction relationship was using for the whole miRNA-mRNA interaction network construction, followed by visualization of this network in cytoscape^65^.

## Supplementary information

Supplementary file1 (XLSX 1157 kb)

Supplementary file2 (XLSX 1208 kb)

Supplementary file3 (PDF 1564 kb)

## Data Availability

The raw sequence data in this study have been deposited in the Genome Sequence Archive in BIG Data Center, Beijing Institute of Genomics (BIG), Chinese Academy of Sciences, with accession numbers CRA001801, CRA001801 that are publicly accessible at https://bigd.big.ac.cn/gsa.
