# Supplementary material for: Degradome, small RNAs and transcriptome sequencing of a high-nicotine cultivated tobacco uncovers miRNA’s function in nicotine biosynthesis
Source: Sci Rep. 2020 Jul 16;10:11751. doi: 10.1038/s41598-020-68691-y (PMC7366715; doi:10.1038/s41598-020-68691-y)
Supplement: Supplementary file 3 — Supplementary file3 (PDF 1564 kb) [file 41598_2020_68691_MOESM3_ESM.pdf]

Degradome, small RNAs and transcriptome sequencing of a high-nicotine cultivated tobacco uncovers miRNA's function in nicotine biosynthesis

Jingjing Jin[1,*], Yalong Xu[1,*], Peng Lu[1], Qiansi Chen[1], Pingping Liu[1], Jinbang Wang[2], Jianfeng Zhang[1], Zefeng Li[1], Aiguo Yang[3], Fengxia Li[3,#], Peijian Cao[1,#]

[1]China Tobacco Gene Research Center, Zhengzhou Tobacco Research Institute of CNTC, Zhengzhou 450001, China;

[2]China Tobacco Science & Technology Information Center, Zhengzhou Tobacco Research Institute of CNTC, Zhengzhou 450001, China;

[3]Tobacco Research Institute, Chinese Academy of Agricultural Sciences, Qingdao 266101, China;

[*]These authors contributed equally;

[#]Corresponding authors: Peijian Cao, peijiancao@163.com

Fengxia Li, lifengxia@caas.cn

Supplementary Figure S1

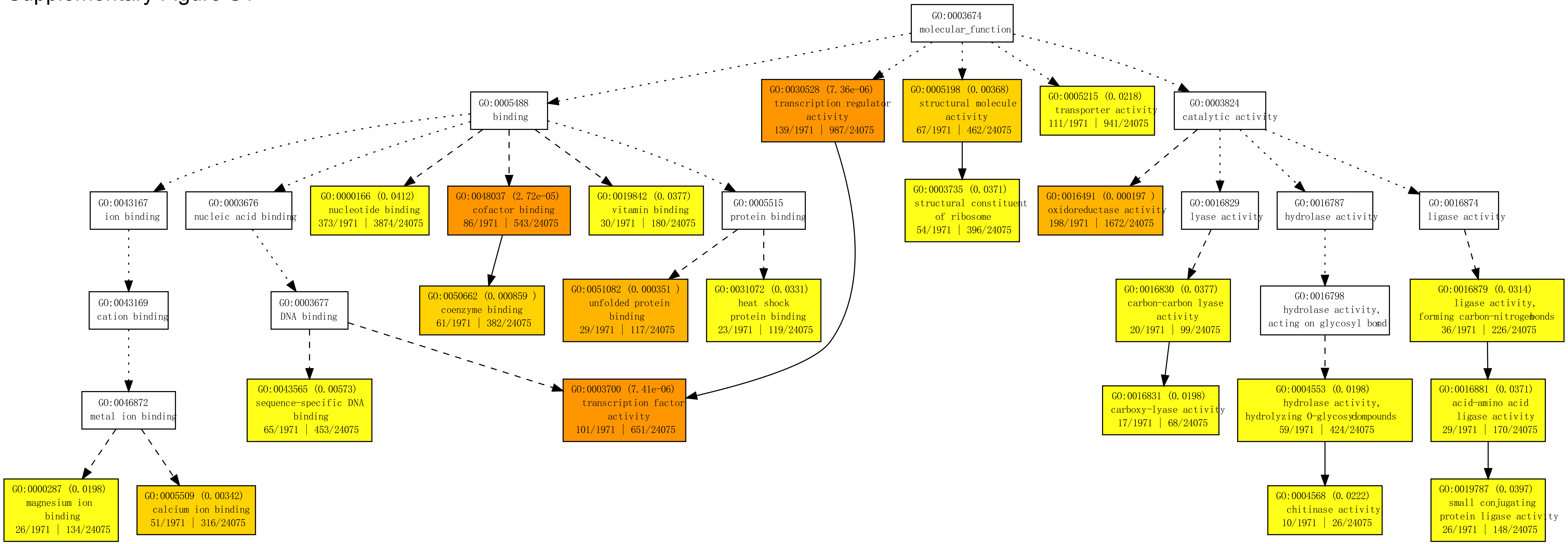

Supplementary Figure S2

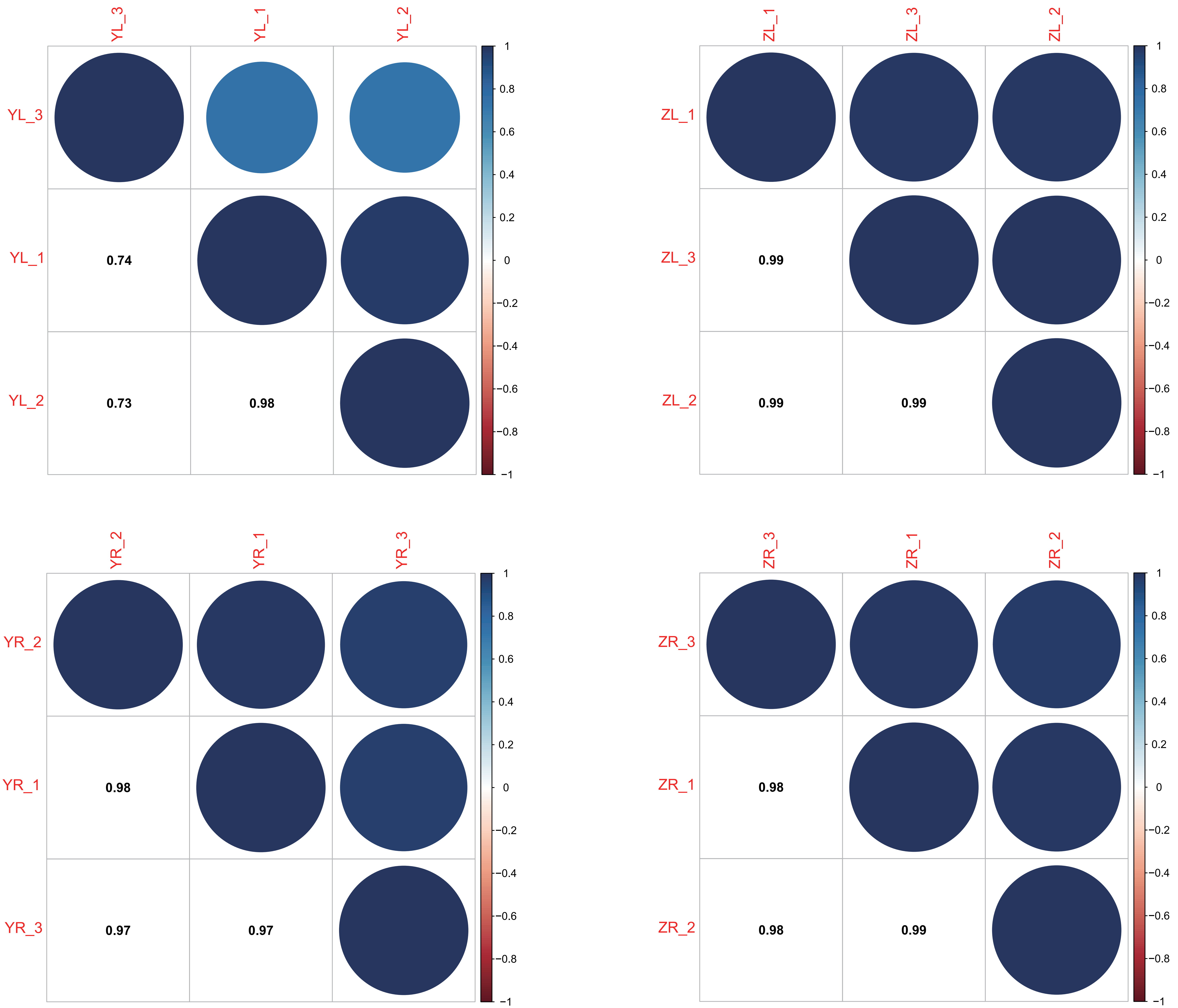

Supplementary Figure S3

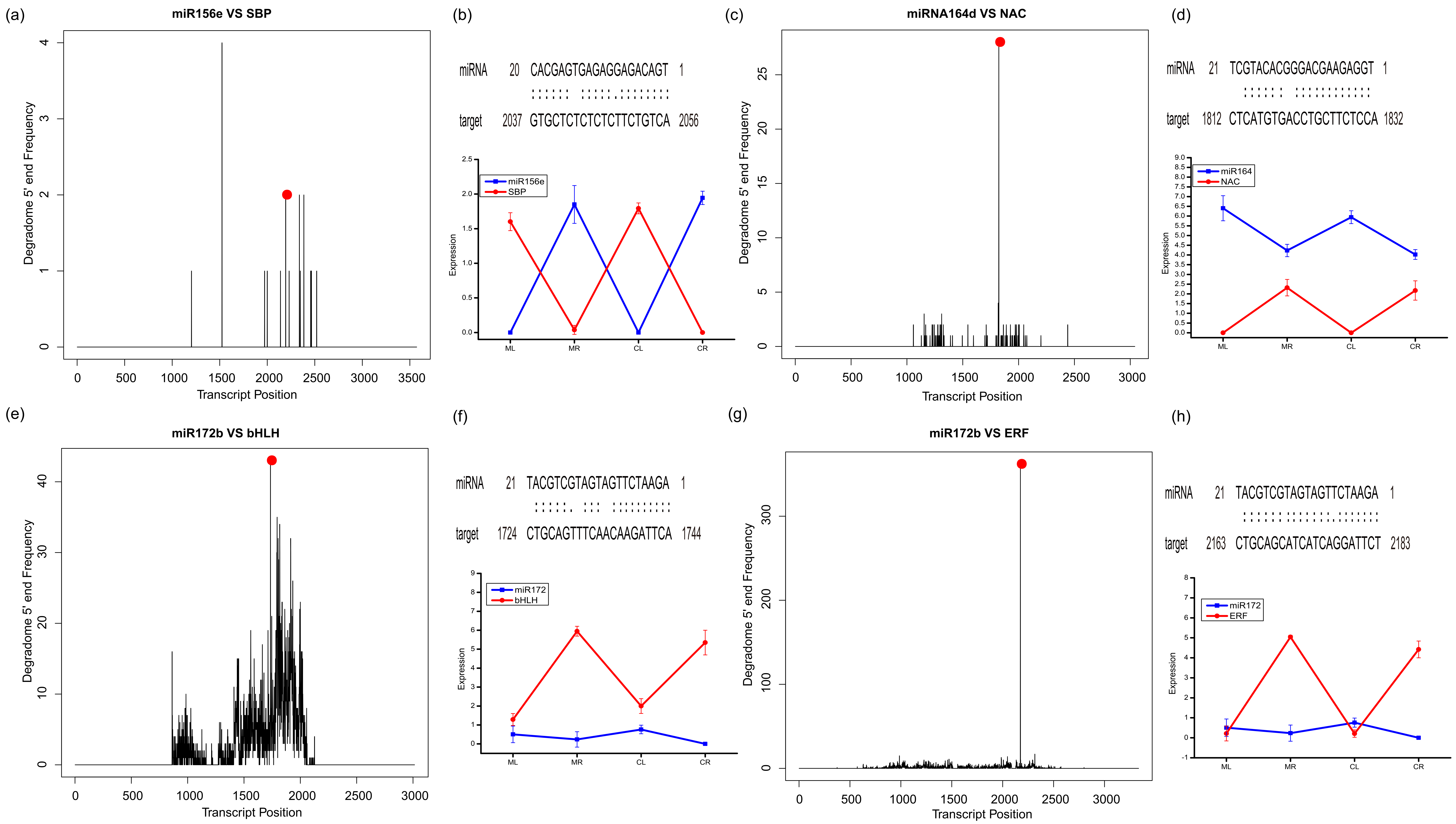

Supplementary Figure S4

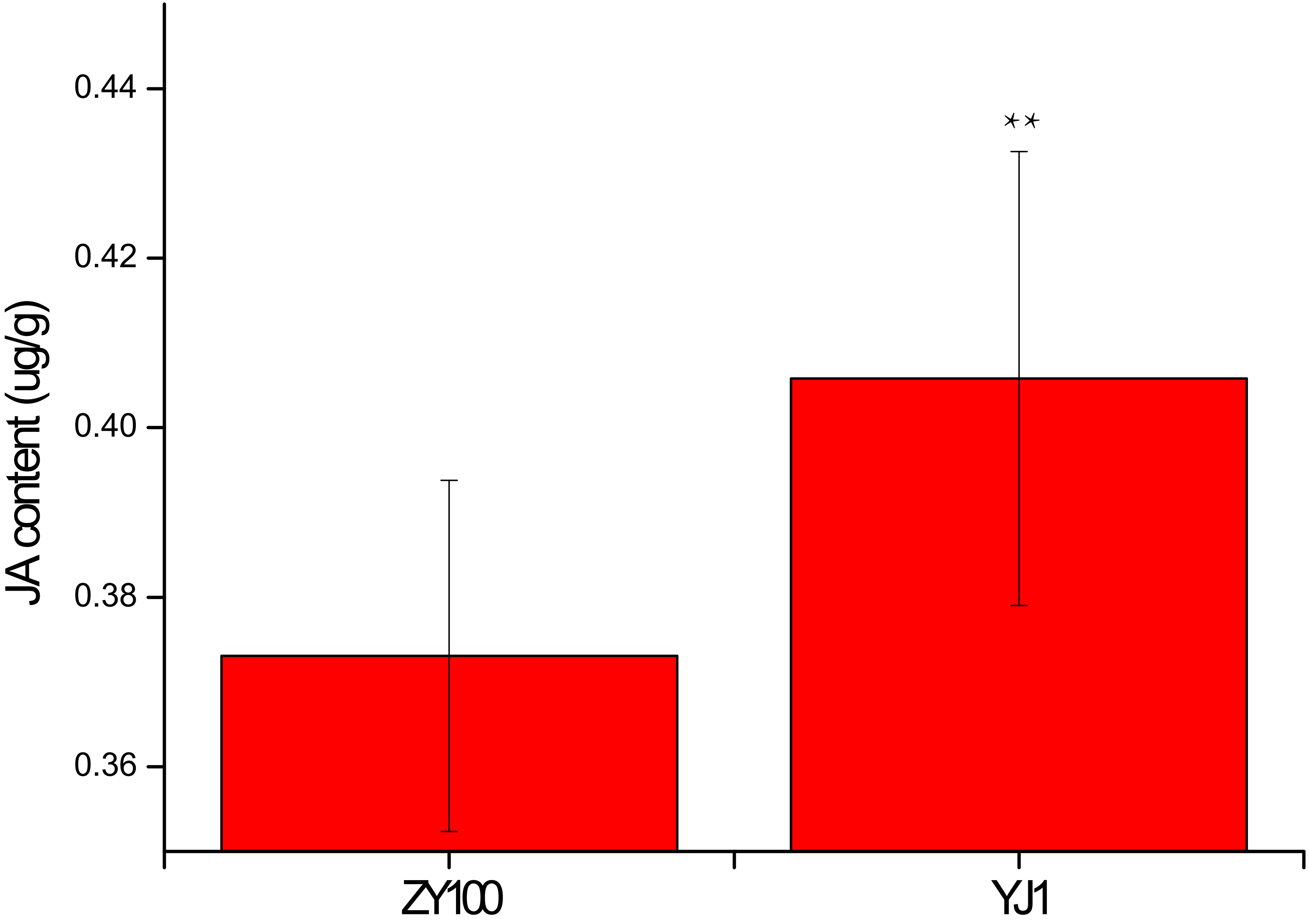

Supplementary Table S1

Family	Arabidopsis thaliana	Vitis vinifera	Glycine max	Oryza sativa	Zea mays	Solanum lycopersicum	Solanum tuberosum	Populus trichocarpa	other species
nta-miR159	Y	Y	Y	Y	Y	Y	Y	Y	
nta-miR162	Y	Y	Y	Y	Y	Y	Y	Y	
nta-miR166	Y	Y	Y	Y	Y	Y	Y	Y	
nta-miR167	Y	Y	Y	Y	Y	Y	Y	Y	
nta-miR168	Y	Y	Y	Y	Y	Y		Y	
nta-miR171	Y	Y	Y	Y	Y	Y	Y	Y	
nta-miR393	Y	Y	Y	Y	Y	Y	Y	Y	
nta-miR396	Y	Y	Y	Y	Y	Y	Y	Y	
nta-miR408	Y	Y	Y	Y	Y	Y	Y	Y	
nta-miR827	Y			Y	Y	Y	Y	Y	
nta-miR1623									gga-miR-1623
nta-miR4376									gma-miR4376a-3p
nta-miR6145									nta-miR6145e
nta-miR6149							Y		
nta-miR7972									rgl-miR7972

Supplementary Table S2

miRNAID	annotation	Seqid(chromosome)	Start position	End position	strand	Mature sequence
miRNA-precursor_679	NovelmiRNA-572	Nitab4.5_0001410	98549	98641	-	CGAUCUGGGUUCGUAUCAAAC
miRNA-precursor_569	NovelmiRNA-479	Nitab4.5_0001059	639579	639677	-	ACGCUGACUGGCGCAUUGAGC
miRNA-precursor_786	NovelmiRNA-663	Nitab4.5_0001730	460728	460826	+	ACGCUAACUGGCGCAUUGAGC
miRNA-precursor_1389	NovelmiRNA-1150	Nitab4.5_0004869	43783	43856	-	AUUGUUAUAUGUUGCACUGGCC
miRNA-precursor_23	NovelmiRNA-22	Nitab4.5_0000014	242877	243022	+	UGGAUGUUAUCUCAGUGUUGG
miRNA-precursor_187	NovelmiRNA-166	Nitab4.5_0000222	138133	138252	-	CGUUUGUGCGUGAAUCUGACA
miRNA-precursor_1593	NovelmiRNA-1316	Nitab4.5_0006503	82723	82843	+	CGUUUGUGCGUGAAUCUGACA
miRNA-precursor_60	NovelmiRNA-57	Nitab4.5_0000042	2206936	2207063	-	AGAGAGGCUGUUUCCGAUAGACCA
miRNA-precursor_87	NovelmiRNA-81	Nitab4.5_0000065	1622605	1622683	+	CUGUGGUCGUGAAGCACUGAC
miRNA-precursor_1632	NovelmiRNA-1348	Nitab4.5_0006950	91308	91411	+	AUCAUGUUAUCCCUUUGGACU
